# Construction and Demolition Waste Management Research: A Science Mapping Analysis

**DOI:** 10.3390/ijerph19084496

**Published:** 2022-04-08

**Authors:** Nehal Elshaboury, Abobakr Al-Sakkaf, Eslam Mohammed Abdelkader, Ghasan Alfalah

**Affiliations:** 1 Construction and Project Management Research Institute, Housing and Building National Research Centre, Giza 12311, Egypt; nehal.elshabory@hbrc.edu.eg; 2 Department of Building, Civil and Environmental Engineering, Concordia University, Montreal, QC H3G 1M8, Canada; 3 Department of Architecture & Environmental Planning, College of Engineering & Petroleum, Hadhramout University, Mukalla 50512, Yemen; 4 Structural Engineering Department, Faculty of Engineering, Cairo University, Giza 12613, Egypt; eslam_ahmed1990@cu.edu.eg; 5 Department of Architecture and Building Science, College of Architecture and Planning, King Saud University, Riyadh 145111, Saudi Arabia; galfalah@ksu.edu.sa

**Keywords:** construction and demolition waste, waste management, bibliometric search, scientometric analysis, science mapping, holistic review

## Abstract

Construction and demolition waste treatment has become an increasingly pressing economic, social, and environmental concern across the world. This study employs a science mapping approach to provide a thorough and systematic examination of the literature on waste management research. This study identifies the most significant journals, authors, publications, keywords, and active countries using bibliometric and scientometric analysis. The search retrieved 895 publications from the Scopus database between 2001 and 2021. The findings reveal that the annual number of publications has risen from less than 15 in 2006 to more than 100 in 2020 and 2021. The results declare that the papers originated in 80 countries and were published in 213 journals. Review, urbanization, resource recovery, waste recycling, and environmental assessment are the top five keywords. Estimation and quantification, comprehensive analysis and assessment, environmental impacts, performance and behavior tests, management plan, diversion practices, and emerging technologies are the key emerging research topics. To identify research gaps and propose a framework for future research studies, an in-depth qualitative analysis is performed. This study serves as a multi-disciplinary reference for researchers and practitioners to relate current study areas to future trends by presenting a broad picture of the latest research in this field.

## 1. Introduction

The building and construction industry utilizes enormous natural resources and produces much waste. Construction and demolition waste (CDW) refers to solid waste generated in the building and construction industries [1]. Tchobanoglous et al. [2] described demolition waste as waste generated from demolished structures. Construction waste, on the other hand, is generated during the construction and renovation of buildings. CDW is produced from the construction, renovation, and demolition operations such as civil works, site clearance, road construction, land excavation or grading, and demolition activities [3]. Floods, earthquakes, and hurricanes are all examples of environmental catastrophes that generate massive amounts of CDW [4]. Rock, masonry, asphalt, metals, sand, plastics, asbestos, plasterboard, and cardboard are among the most typical CDW material profiles [5]. CDW accounts for more than 30% of the total solid waste generated around the globe [6]. Significant quantities of CDW have been generated annually across the world. For example, China is the world’s largest CDW producer, with around 2300 million tons in 2019 [7]. Meanwhile, the United States and European Union produced around 600 and 834 million tons of CDW in 2018, respectively [8,9]. Large proportions of CDW are recyclable. On the other hand, there might be a small percentage of toxic materials that have negative consequences for humans and the environment. As a result, there is a compelling need to reduce CDW generation and its environmental implications [10].

Solid waste is produced by households, commerce, the building and construction industry, and other industries [11]. According to the “Global Waste Management Outlook” that was issued by the International Solid Waste Association (ISWA) and the United Nations Environmental Program (UNEP), about 85% of the total generated solid waste worldwide is disposed of in landfills, with very low reuse and recycling rates [12]. Almost 70–80% of CDW is dumped in landfills while only 3% is recycled in Beijing, China [13]. The situation gets worse because of the prevalence of illegal dumping in recent years [14]. The illegal dumping of such waste hinders sustainable urban growth because it is associated with negative economic, social, and environmental consequences [15,16]. The need of reducing, reusing, and recycling CDW to reduce the strain on landfills and improve waste diversion practices, has fueled the sustainability movement from both governmental and industrial viewpoints [6]. CDW management is an interdisciplinary topic that addresses complex issues from the engineering, management, technological, and policy perspectives and contributes to the circular economy [17,18,19]. 

Due to a growing interest in dealing with construction and demolition challenges around the world, there have been a lot of scholarly publications in recent years. These papers are usually divided into two categories: (1) investigating CDW management from a broad viewpoint, including identification of significant themes and analysis of research trends in management and recycling [20]; and (2) examining a specific research area of quantification methods [21], prediction models [22,23], transportation [24], recycled aggregates [25,26], and treatment methods [27]. However, it is unclear how this research topic has progressed over time and whether the adopted management disciplines have changed since prior studies. There has been a dearth of thorough and systematic literature reviews that examine CDW management research to follow up on the studies undertaken by [10,28]. 

Proper implementation of the CDW management plan contributes to the three pillars of sustainability (i.e., economic, social, and environmental aspects). It provides many benefits to contractors in the form of cost savings, to the general public and authorities in the form of improved public health and reduced social problems caused by waste accumulations, and to the environment in the form of resource efficiency [29]. However, the success of CDW management is hindered by the conflicting concerns of the two main stakeholder groups involved in the process. Authorities, the general public, and non-governmental organizations make up the first stakeholder group, which is primarily concerned with reducing the quantity of landfilled waste. The second category includes subcontractors, main contractors, and project clients, who are primarily concerned with the economics and revenues of executing waste management, rather than the impact of CDW on the environment. In this context, a comprehensive content analysis of publications in relation to CDW management would be critical for assisting all stakeholders in comprehending the latest practices and advances, as well as serving as a medium for motivating new research and practical ideas [30]. 

Bibliometric and scientometric analyses, often known as “science mapping”, are used to investigate the trends and gaps in a particular study topic [31]. Bibliometric studies have been used by several academics to depict or map the structure and evolution of literature. The approach is gaining popularity as a research tool for examining the knowledge domain or visualizing networks to offer a more comprehensive view of the subject [32]. Jin et al. [33] and Xu et al. [34] stated that a scientific mapping approach might be included in a holistic review by adding an in-depth qualitative analysis. This analysis might lead to a new research framework directing future scholarly work. 

Li et al. [35] conducted a bibliometric study on solid waste reuse and recycling in developing and developed countries from 1992 to 2016. Every five years, research directions were investigated, and social network analysis was performed to examine author collaborations and keyword co-occurrence. Jin et al. [29] applied a scientific mapping technique to review CDW management research from 2009 to 2018. The study identified the most significant and influential authors, publications, journals, and countries using bibliometric search, scientometric analysis, and qualitative discussion. The qualitative analysis summarized the main research areas, discussed research gaps, and proposed a framework for future research. Wu et al. [10] conducted a holistic review to examine CDW publications between 1994 and 2017. This research revealed the most active authors, organizations, and countries in this domain. Furthermore, it clustered the CDW research based on a keyword cluster analysis. Finally, it presented the current status and future potential directions of research.

The novelty of this research study is: (1) applying the bibliometric and scientometric analyses to perform the holistic review in the CDW management domain. This approach could minimize subjectivity and biases in performing review-based studies [36], (2) providing a comprehensive analysis of articles published from 2001 to 2021, and (3) extending the science mapping approach in waste management review with an in-depth qualitative analysis that identifies the current research status and emerging trends. The research objectives could be identified as follows: (1) conducting a holistic review to identify time and geographical distribution, journals, citations, authorship, keywords, and co-citations of references in the CDW management research field, (2) analyzing the key research themes in this domain, and (3) identifying the current research gaps and providing a framework to guide future research paths. 

## 2. Methodology 

**Figure 1:**
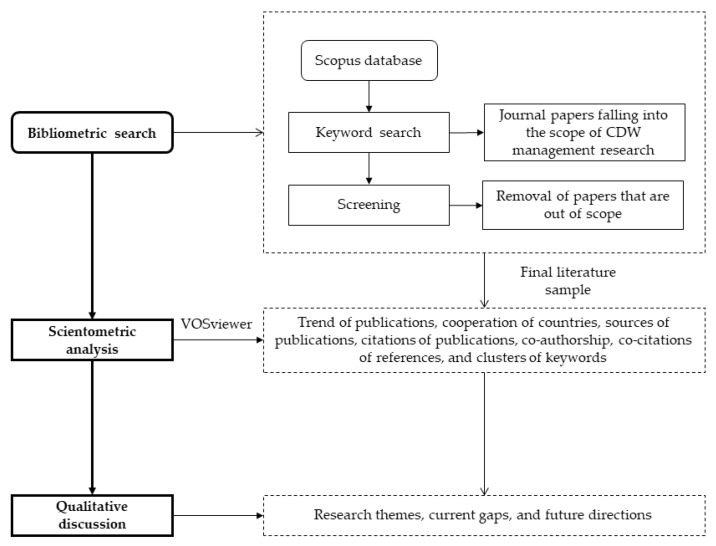
Workflow of the research study.

This review-based study evaluates the most recent research articles that have been published in Scopus (i.e., 2001–2021) in the domain of CDW management. It employs a holistic analysis approach to better understand the study area and remove biased findings [37]. Figure 1 illustrates the research workflow, which includes bibliometric search, scientometric analysis, and qualitative discussion.

### 2.1. Bibliometric Search 

The bibliometric analysis provides a thorough overview of the study domain by analyzing relevant research studies [32]. Scopus is one of the largest and most popular search engines, with regional and global coverage of academic outputs. Compared to other databases such as Web of Science, it includes more recent articles and journals [38]. It has been widely used as a data source for bibliometric analysis in review papers [39]. Therefore, the Scopus database is used in this study to conduct a bibliometric search of CDW management papers. The search was conducted on the 23rd of November 2021. The literature search begins with the following keywords: TITLE-ABS-KEY (“CDW” OR “C&D waste” OR “C&D wastes” OR “construction and demolition waste” OR “construction waste” OR “demolition waste” AND “waste management”). The targeted research and review papers are published between 2001 and 2021 in English. Because conference papers do not contain as much information as journal articles, they were omitted [40]. Further screening is performed by scanning titles and abstracts to filter publications that are either out of scope or do not focus on CDW management.

### 2.2. Scientometric Analysis 

The scientometric analysis is used to map the current state of knowledge and the evolution of a research domain. It entails the co-occurrence of journals, keywords, active countries, citation and co-citation analysis, and bibliographic coupling of researchers and documents [41]. Scientometric searches were initially accomplished by manual selection and categorization of articles [42]. With the rapid advancement of technology, multiple science mapping software applications are now accessible to graphically depict various elements of scientific research [43]. VOSviewer, CiteSpace, and Bibexcel are a few examples of these software applications. Because of its aptitude for knowledge mining and visualization of vast networks, VOSviewer (version 1.6.15) is utilized in this study to undertake scientometric analysis [44]. 

VOSviewer software use jargon such as clusters, items, networks, and links. It can display maps in three distinct formats: overlay, network, and density visualizations. Nodes in network visualization indicate many elements such as authors, keywords, publications, or countries. The size of a node is directly proportional to an item’s relevance. The color of a node denotes the cluster to which it belongs. The lines connecting nodes represent intellectual links/connections. The distance between two nodes reflects how closely they are linked. Because the items are arranged into clusters, the overlay view mimics the network visualization. A scale bar is used to calculate the scores of clusters/items. The density graphic depicts the frequency of occurrence of various items [45].

VOSViewer has been used in many research studies in the field of construction engineering and project management to aid in the literature review of various topics, such as system dynamic applications [46], building information modeling (BIM) [47], and construction safety management technologies [48]. VOSViewer is used in this study to fulfill the following goals: (1) import the Scopus literature; (2) display and evaluate the most important journals, publications, scientists, and countries in the CDW management research community; and (3) investigate the research keywords and their interrelationships.

### 2.3. Qualitative Analysis

Following the bibliometric search and scientometric analysis, the final phase is conducting an in-depth qualitative discussion of the content of literature samples based on a four-fold philosophical framework. Previous studies have employed the four-fold philosophical framework of axiology, methodology, ontology, and epistemology to provide a systematic understanding of the research topic [49]. According to the findings of Pan et al. [50], when combined with the objectives of this paper, axiology, methodology, as well as ontology and epistemology aspects are interpreted by defining the objective, research method, and definition of CDW management. In other words, the functional dimension represents axiology, which is concerned with the “function and value of CDW management”. The methodological dimension refers to methodologies that demonstrate “how research on CDW management is conducted”. The ontological and epistemological cluster denotes “what CDW management is and how to understand it” in this context. This analysis is undertaken to review the important research subjects, identify research gaps, and recommend future research paths in CDW management.

## 3. Results and Findings 

Scopus provides a list of 996 journal articles based on a keyword-based bibliometric search. Full list of the utilized database can be found in the Supplementary Materials. Some research studies that extended beyond the construction and demolition sectors are omitted from the literature review during the screening process. For example, Moura et al. [51] and Cai et al. [52] focused on other types of waste (e.g., municipal solid waste). Some studies addressed construction management research in general [46,53]. Other studies such as Nasab et al. [54] intended to investigate additional sustainability issues (e.g., building carbon footprints). Finally, a total of 895 papers are exported as plain text files, including their authors, affiliations, title, publication year, journal, citations, abstract, keywords, and references for further processing and analysis.

### 3.1. Results of Scientometric Analysis

#### 3.1.1. Number of Publications

**Figure 2:**
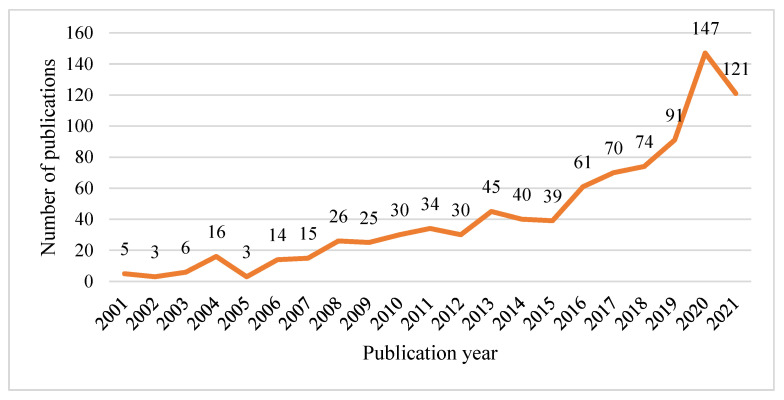
Time distribution of publications from 2001 to 2021.

The search yielded 895 CDW-related publications between 2001 and 2021. Figure 2 illustrates the general trend in CDW management research outputs during the last twenty years. The annual number of publications has been steadily growing since 2006, rising from less than 15 in 2006 to more than 100 in 2020 and 2021. This affirms that the research community has become more interested in researching CDW management in recent years.

#### 3.1.2. Sources of Publications

**Figure 3:**
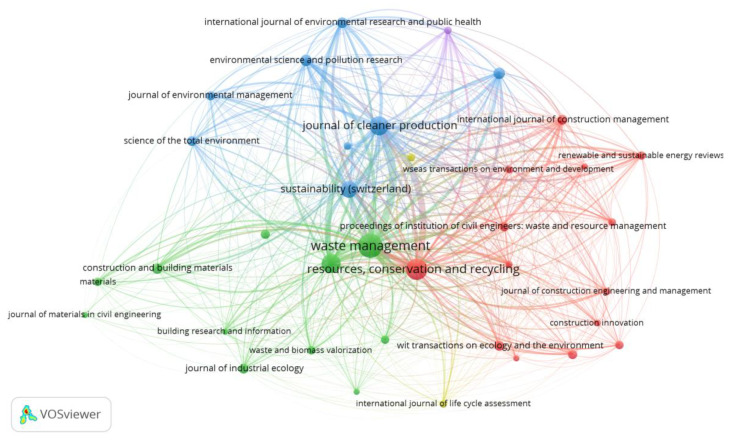
Mapping of journals in CDW management research.

Scholars can identify journals suitable for publication outputs by analyzing the publication sources [41]. The 895 papers are published in 213 different journals, demonstrating a wide range of geographical locations and disciplines. In VOSViewer, the minimum number of published articles and number of citations is set to 3 and 30, respectively [29]. It is found that 36 out of 213 journals matched the criteria. Figure 3 illustrates the most productive journals where the selected CDW management journal articles are published. The font and node sizes graphically depict the number of publications in each journal, with bigger font and node sizes signifying more publications. The connecting lines show how closely journals are related in terms of mutual citations.

As summarized in Table 1, the influence of journals in the CDW management domain is quantified using five important variables, including the number of publications, average publication year, total citations, average citations, and average normal citations. Citation is one of the most popular ways to discover significant research outputs on a subject. The average normal citation index of a journal is calculated by dividing the total number of citations by the average number of citations published each year. The normalization corrects the misunderstanding that older articles acquire more citations than newer publications [55]. The average normal citation index is used to identify fruitful journals in this research.

More than half of the CDW-related articles are published in five journals. For instance, the Waste Management Journal releases more than 100 papers, while Resources Conservation and Recycling, Waste Management and Research, and Journal of Cleaner Production publish above 50 articles. These journals have all made major contributions to the research community in CDW management. The most recent papers appeared in Materials and the International Journal of Environmental Research and Public Health. On the contrary, Construction Management and Economics, as well as Management of Environmental Quality journals, are not active in this study arena for a long time. While the number of publications and total citations indicators are usually highly correlated, average citations and average normal citations may be unrelated to the first two metrics. In other words, a journal that is productive in terms of the number of publications or total citations may not have the highest average citation or normalized average citation per article. For instance, the Waste Management journal is characterized by the highest number of publications (i.e., 129) and total citations (i.e., 7522). Meanwhile, the Building and Environment Journal is associated with the highest average citations (i.e., 90.38), indicating the strongest impact in terms of research outputs. In terms of average normal citations, the Journal of Cleaner Production has the highest average yearly influence (i.e., 2.2). Table 1 also shows other journals that are not among the top in terms of output yet contribute significantly based on average citations. Journal of Materials in Civil Engineering, Construction Management, and Economics, and Renewable and Sustainable Energy Reviews are among these journals. 

#### 3.1.3. Co-Authorship Analysis 

The co-authorship analysis aims at determining the most prolific scholars and organizations in a given research field. In academic research, researchers can increase their productivity by becoming aware of potential possible partnerships and collaborations in their fields [56]. The minimum number of publications and citations of an author for this authorship analysis are 5 and 30, respectively [29]. The selection criteria are satisfied by 74 out of 2121 scholars in the literature sample. 

Some of the most influential researchers are listed in Table 2. The number of published papers, average citations, average publication year, total citations, and average normal citations are the five primary quantitative metrics of scholars. Lu W. and Shen L. rank first in terms of the total number of articles (i.e., 43) and average citations (i.e., 114.33), respectively. Recently active academics such as Bao Z., Xue F., Chi B., and Hao J. whose publications are normally around 2020, can be found using the average publication year of scholars. Yuan H. is the most productive author in the field of CDW management, with substantially more total citations (i.e., 2206) than the other authors. The normal citation analysis shows how influential scholars are on an annual basis. Despite Shen L., Yuan H., and Zillante G. do not have the largest number of publications, they have made considerable contributions to CDW management research as measured by their annual impact. The average normal citations are the same for several scholars in the same cluster. For example, the average normal citations of Yu A.T.W. and Wu Z. are 1.70. This means that both scholars contributed equally to the research field. In addition, several groups of researchers differ with respect to the average normal citations, indicating collaboration of these researchers with other groups or working alone to develop novel collisions. Despite having only six publications, Kabirifar K. has the greatest average normal citations (i.e., 5.61).

#### 3.1.4. Citations of Publications

The most influential journal articles during the previous twenty years are examined after setting a minimum citation of 50 [29,49]. As a result, 199 articles are chosen out of a total of 895. The most influential publications, as assessed by average normal citations, are depicted in Table 3. It summarizes further information on the top ten articles, including their entire tile, source, publication year, total citations, and average normal citations. 

One of the early studies to examine architects’ approach towards construction waste reduction and investigate sources of waste, waste reduction practices, and responsibilities and barriers of architects is Osmani et al. [57]. Jin et al. [29]’s review-based study has achieved the sixth-highest citation rate in the last twenty years. The research trends, including the study of qualities of recycled products, waste quantification methods, and waste diversion practices, are currently being researched. Marinković et al. [58], the highest cited paper, focused on determining the structural applications of recycled aggregate concrete and comparing the environmental impact of concrete made from virgin and recycled aggregates. Liu et al. [59] gained the most yearly-based attention in the academic community. The research simulated the economic, social, and environmental aspects of various CDW disposal methods using the system dynamics approach.

#### 3.1.5. Active Countries

**Figure 4:**
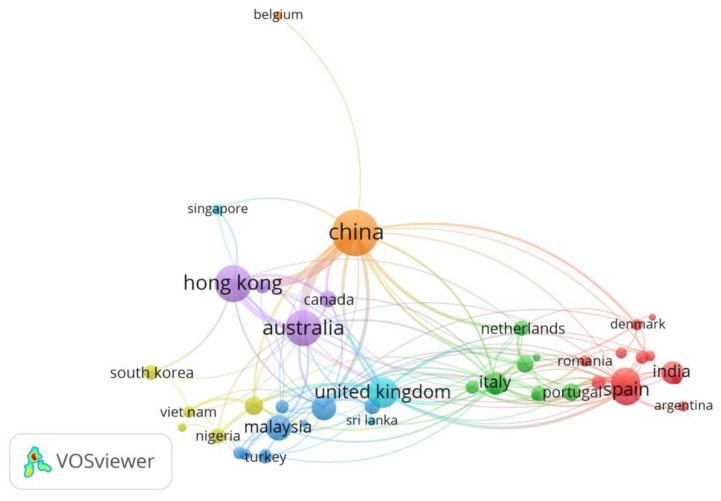
Mapping of the active countries in CDW management research.

In this study, the contributions of different countries to the global research community are analyzed, with the minimum number of articles and citations set at 3 and 30, respectively [29]. As a consequence, 47 out of 80 countries are short-listed. The publications originated from 47 countries, including 21 from Europe, 16 from Asia, 3 from Africa, 2 from Oceania, 2 from North America, and 3 from South America. It is discovered that 22 countries (47%) produced fewer than 10 articles, while 25 countries published the remaining papers (53%). The active countries that have been engaged in CDW management research in the last twenty years are presented in Figure 4.

A country with a larger node has more opportunities to collaborate with scholars from other nations. According to their node sizes and connecting lines with other countries, the following countries have contributed to the research community: China, Hong Kong, Australia, Spain, and United Kingdom (see Figure 4). China has a close collaborative relationship with Hong Kong and Australia. Spain is not in the same cluster as the preceding two, but it has a collaborative relationship with Argentina, which does not collaborate with any other country. The network shows that the countries with the highest publications collaborate. Furthermore, the network exemplifies the collaboration growth among academics in developing and developed countries. The results demonstrate that the sizes of nodes for Malaysia and the United Kingdom are similar, although Malaysia only publishes a third of the United Kingdom’s articles. It demonstrates that Malaysia and United Kingdom are all working hard to establish a collaborative network with experts from other parts of the world. 

Table 4 provides more quantitative data, such as the number of published papers, average publication year, total citations, average citations, and average normal citations. In terms of the number of publications, China, Hong Kong, Australia, Spain, and the United Kingdom are the top five most productive countries, with 189, 116, 111, 77, and 75 articles, respectively. Because both developed and developing nations are rated among the top five countries, academic contributions are not solely dependent on economic progress. Furthermore, the articles have a worldwide reach because they are scattered across three continents: Europe, Asia, and Oceania. 

With respect to the total citations, Hong Kong and Chinese scholars lead the other countries, followed by Australia, the United Kingdom, and Spain. The papers published by Hong Kong, China, Australia, the United Kingdom, and Spain receive 6879, 6624, 3790, 3451, and 3196 citations, respectively. Although some countries publish fewer publications, they have more overall citations. For example, Spain has only 77 published articles with 3196 total citations. Meanwhile, according to the time trend analysis, Slovenia, Norway, and Taiwan began researching CDW far earlier, with the main research outputs reported around 2011, whereas the main publications in Vietnam, Luxembourg, and Saudi Arabia were produced in 2019. With respect to the average citations, Serbia, Ireland, Singapore, Denmark, and Germany have the highest values of 95.75, 91.75, 68.29, 64.5, and 59.85, respectively. According to the average normal citations, Vietnam, Ireland, Denmark, Singapore, and Serbia have had a greater annual influence on the research community. Assessing the economic viability of waste recycling, the benefits associated with waste management in general, and recycling in particular, and the composition of waste have been among the hot research topics in Vietnam [65], Ireland [66], Denmark [67], Singapore [68], and Serbia [58]. 

#### 3.1.6. Co-Citations of References 

Co-citation analysis is the inverse of bibliographic coupling, concentrating on publications rather than researchers. It calculates related publications by counting the number of times these papers are cited in other articles together [69,70]. A criterion of at least 15 co-citations is imposed, and 16 articles are extracted. Table 5 shows the top five most co-cited references due to space constraints. The majority of the cited references are review articles, which often earn more citations than research publications [71].

#### 3.1.7. Co-Occurrence of Keywords

**Figure 5:**
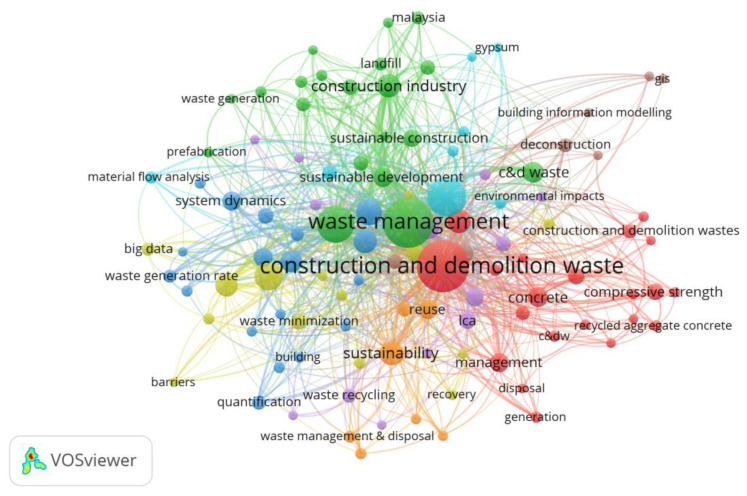
Co-occurrence of keywords in CDW management research.

Keywords describe the focus area within a certain domain. The relationships and conceptual arrangement of research topics are represented by a network of keywords [55]. Therefore, the analysis of the keywords in articles aids in identifying the major research themes, hotspots, and gaps [76]. In this research, the “author keywords” and “fractional counting” are employed in VOSViewer analysis, as recommended by [77] and [56]. The threshold value for the frequency of keyword occurrence is set at 5. About 108 out of 2126 keywords satisfy the threshold value, with broad terms such as waste, solid waste, construction, and demolition waste, CDW, construction waste, demolition waste, construction, demolition, deconstruction, building construction, construction project, construction industry, construction sector, construction materials, urban, city, road, and country names (e.g., China, Shenzhen, and Hong Kong) being deleted. Additionally, certain terms with similar semantic meanings are merged, such as building information modeling (BIM) against building information modeling and C&D waste management versus construction and demolition waste management. Co-occurrence analysis of keywords in CDW management research is illustrated in Figure 5. The keywords are represented by the node sizes, distances between nodes, and connecting lines among keywords. Different node colors represent different clusters of keywords groups. Waste management & disposal and environment keywords are closely connected inside the same cluster. Furthermore, keywords from distinct clusters, such as waste management and recycling, may be tightly connected. This suggests that co-occurrence networks can provide influential and high-frequency keywords while also defining the research scope of a specific field.

Table 6 summarizes further quantitative measures of the top five keywords: occurrences, average publication year, average citations, and average normal citations. The average publication year illustrates how current a term is in the area of CDW management. For example, studies on resource recovery were mostly published around 2013, indicating that this topic was investigated quite early. On the other hand, papers on waste recycling, urbanization, and environmental assessment were published in 2017–2019. This shows that these topics piqued academics’ interest in recent years and might represent future study possibilities. According to the average citations, the following keywords have garnered more attention in the research community: review, urbanization, waste recycling, resource recovery, and environmental assessment. The average normal citation values indicate the interest of the CDW management research community. For instance, the review term has the highest average normal citation compared to other keywords.

### 3.2. Findings of Qualitative Assessment 

Based on the cluster analysis of keywords in Figure 5, it can be interpreted that the research themes could be divided into seven clusters: (1) waste quantification approaches (green cluster), (2) analysis methods of waste management (blue/ purple clusters), (3) environmental impacts of waste (orange cluster), (4) performance and behavior of waste (red cluster), (5) waste management plan (cyan color), (6) waste diversion practices (yellow cluster), and (7) emerging technologies in waste management (brown cluster). However, absolute reliance on the software to cluster the research areas is ineffective. Therefore, this research conducts an in-depth qualitative assessment in order to describe emerging research themes, highlight research gaps, and offer a framework for future research directions as shown in the next sub-sections.

**Figure 6:**
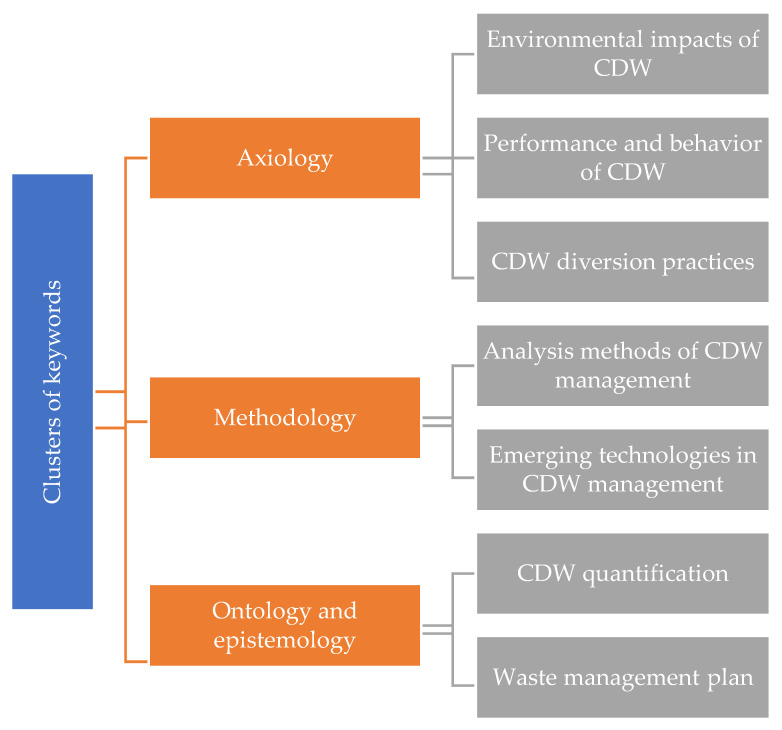
Clusters of keywords based on the four-fold philosophical framework.

This paper categorizes the identified themes for further interpretation based on the four-fold philosophical framework, as shown in Figure 6. The four philosophical aspects of axiology, methodology, ontology, and epistemology are implicitly addressed in all of the keywords. The three themes considered in the functional dimension are (1) associated environmental impacts, which investigate the environmental implications of CDW management; (2) related performance and behavior, which examine the performance and behavior of waste and recycled products; and (3) adopted diversion practices, which refer to the CDW disposal practices. The two themes elaborating on the methodological dimension are (1) analysis methods, which define the research methods used for data collection and analysis for studying CDW management; and (2) emerging technologies, which study the application of information technology in this research field. The two themes describing the ontological and epistemological dimensions are (1) waste quantification, which involves estimating the volumes of accumulated and generated waste to adopt the appropriate waste management strategy; and (2) waste management plan, which discusses the waste minimization and prevention methods to ensure effective decision-making in the early project stages. The seven mainstream research themes are described as follows:

#### 3.2.1. Waste Quantification

The quantity of CDW must be estimated for building a successful waste management plan. Quantification at the project level can aid project managers in arranging on-site stockpiling, adjusting material acquisition schedules, and calculating the waste disposal cost and recycling benefit. Meanwhile, quantification at the regional level entails estimating the overall waste generation from all projects in a given area. This can help decision-makers develop more realistic regulations, allocate labor and truck resources, and plan for the construction of new landfills [78]. There are two approaches for quantifying the volumes or rates of waste generation at the site: soft and hard measure methods. Interviews, questionnaire surveys, and statistical data are among the soft measure methods. While hard measurement methods include the material flow analysis approach as well as sorting and weighing of waste materials [79]. Waste generation rate and reuse/recycling are examples of the keywords in this cluster. According to the keywords revealed in the cluster analysis, this cluster is mostly concerned with the following issues: (1) quantifying CDW generation, for instance, Attia et al. [4] quantified waste using GeoSLAM’s simultaneous localization and mapping (SLAM)-based mobile mapping system in Kafr El Sheikh, Egypt. Moreover, Asgari et al. [80] assessed the quantity of CDW in Tehran, Iran using questionnaire methods; (2) investigating waste generation rates such as Islam et al. [81]’s study which adopted a holistic approach to investigate waste generation rates and compared the acquired data against that in other countries; and (3) developing CDW generation estimation models by the application of machine learning models [24,82].

#### 3.2.2. Analysis Methods of Waste Management

Circular economy, life cycle assessment (LCA), life cycle costing, system dynamics, and cost-benefit analysis keywords belong to this cluster. CDW was traditionally regarded as zero-value materials in a linear economy, and as a result, the majority of waste materials were disposed of in landfills. With increased awareness in recent years, different countries have explored innovative approaches to minimize the use of non-renewable resources. In this context, the circular economy has emerged as a promising strategy for achieving long-term development and increased resource throughput as well as reducing the negative environmental impact of waste. This emerging concept attempts to replace the current production and consumption paradigm [83]. However, there is currently a lack of a defined body of knowledge or framework for applying circular economy to CDW management [13]. LCA is beneficial for evaluating the economic and environmental performance of building materials and processes throughout different life cycle stages, including design, construction, operation, and end-of-life. Besides, it is an innovative tool that could be applied to assessing the performance of CDW management by considering the critical aspects that might require improvement actions [84,85]. System dynamics is an empirical tool, and waste management must be studied and analyzed dynamically to avoid misinterpretation in this field area. An example of the application of the system dynamics approach includes management and control of the waste management system so that it has fewer negative consequences for the environment and society [86]. Another example is the examination of the optimum waste disposal charge for long-term sustainability, as well as linkages between economic/social aspects and waste disposal charging fees [87]. This cluster also involves studies related to performing a cost-benefit analysis, including economic and financial analysis, by accounting for the social, environmental, and economic aspects of CDW recycling [88].

#### 3.2.3. Environmental Impacts of Waste

This cluster is concerned with investigating the environmental implications of CDW, which helps authorities and stakeholders make decisions about the collection, treatment, and disposal plans [89]. It incorporates sustainability, environment, waste management, and disposal keywords. CDW has a variety of negative environmental effects, including land usage, landfill depletion, pollution, resource depletion, and so on. Landfilling not only consumes a substantial amount of land but also pollutes the soil. Moreover, because of the presence of CDW leachate, unauthorized dumping may represent a risk to surface and groundwater. Demolition operations not only result in a huge number of surplus construction materials but also squander resources. Meanwhile, the employment of vehicles and machinery in the disposal process may have several negative consequences, including dust and noise pollution [90]. The environmental implications of CDW have been discussed with respect to several topics: (1) waste composition characterization and distribution as existing research studies indicate that CDW stream may comprise different pollutant compositions, such as organic materials and heavy metals. These contaminants would have an impact on the surrounding environment (e.g., soil, groundwater, and water) [91,92]; (2) environmental impact assessment which could be conducted using system dynamics such as the study performed by Marzouk and Azab [73]; and (3) mitigation actions for the pollution caused by landfilled waste, which can be verified by quantifying the total saved energy and avoided emissions by recycling waste. From an environmental standpoint, recycled aggregates have been claimed to be sustainable in areas where natural resources are few or large distances for the transportation of virgin materials are required. As a result, it is crucial to undertake and support environmentally sound decisions for CDW management [89].

#### 3.2.4. Performance and Behavior of Waste

This cluster is concerned with investigating the performance and examining the behavior of waste and recycled products. Keywords in this cluster comprise strength, compressive strength, and mechanical properties. Due to building structure heterogeneity, existing demolition practices that compromise waste uniformity, and the absence of effective treatments for recycled aggregate generation, the composition of CDW is extremely diverse. Therefore, it is of acute importance to undertake the required tests to determine the optimal methodology to deal with waste and recycled products [93]. Studies on the CDW recycled products have been undertaken from different viewpoints, including CDW management performance [26], behavior and properties of CDW or recovered products [94], applications for recyclable waste materials [95], and performance improvements of CDW recycled products [96]. Meanwhile, other research focuses on particular performance-related tests of recycled aggregates (e.g., strength, deformation, tensile strength, stiffness, and durability) [97,98]. Evaluating the type and behavior of waste assists in determining the suitable strategy, while assessing the characteristics of recycled aggregates is a necessity to determine their optimum applications. It was noticed that it is more common to use CDW recycled aggregates as a replacement for natural aggregates in the pavement construction industry if compared to their utilization in the geotechnical applications and construction of buildings [99].

#### 3.2.5. Waste Management Plan

Developing a waste management plan ensures effective decision-making in the project design and planning stages [100,101]. Appropriate waste management strategies can assist to reduce waste generation, promote waste reuse and recycling, and mitigate waste disposed of in landfills [102]. Proper implementation of the CDW management plan adds to the three pillars of sustainability (i.e., economic, social, and environmental aspects). It has several advantages for contractors in terms of cost savings, for the general public and authorities in terms of enhanced public health and decreased social issues caused by waste accumulations, and for the environment in terms of resource efficiency [20]. Solid design and construction management may save up to 40% on waste [103]. This cluster involves solid waste management and resource recovery keywords. Waste can be used as input materials to generate new valuable outputs, known as resource recovery. The goal is to limit waste generation and accumulation, minimize the requirement for landfill space, maximize the value derived from waste, and reduce the utilization of raw resources in the manufacturing process. The following information shall be included in an effective waste management plan [104]: (1) goals for waste recycling, salvage, or reuse, (2) estimated types and amounts of waste at the project site, (3) proposed disposal methods for these materials, and (4) procedures to be followed when dealing with waste.

#### 3.2.6. Waste Diversion Practices

Waste management should obtain the sustainability movement not only from the government but also from the industry, in order to improve waste diversion practices and relieve landfill pressure [105]. More research studies on CDW diversion have been conducted in the last decade, with an emphasis on the internal and external variables that impact the CDW disposal practice. Recovery, waste minimization, barriers, and illegal dumping keywords belong to this cluster. One of the most important external factors, together with the local market and the availability of landfills, is government support [106]. Emerging technologies [107], material procurement [108], sorting strategies [109], and stakeholder factors [110] are some of the key internal factors related to waste management in the construction industry. There are a variety of strategies that could be utilized by a municipality to encourage the diversion of waste from landfills [111]: (1) developing a waste management plan, (2) educating and informing contractors about waste disposal options other than landfills, (3) placing policies to divert waste, (4) including specifications in local construction projects, (5) encouraging green construction and reusing waste materials that have been recovered and reprocessed, (6) adopting waste hauler incentive programs to encourage them to divert more waste products from landfills, and (7) enforcing a waste diversion ordinance. Researchers have identified several key barriers to promoting CDW reuse, recycling, and reduction, including lack of awareness, commitment, supervision, and legal enforcement [112], poor quality of recycled products [113], and immature recycling technology [13].

#### 3.2.7. Emerging Technologies in Waste Management

This cluster discusses the application of information technology in CDW research. It includes construction waste management, BIM, GIS, and optimization keywords. Lack of historical data, inaccuracy in estimation, inconsistencies in reported data, and lack of established platforms for promoting the circularity of recovered waste materials through reusing and recycling are among the issues hindering the application of advanced technology use in waste management [114]. Additionally, existing CDW management tools suffer from insufficient data quality, lack of interoperability with other software, and inability to integrate with the design process [107]. Some academics have reported that the dearth of innovative technology in CDW management is impeding the development of effective management practices in the construction industry. Globally, information technologies are rapidly being used to improve the efficiency of CDW management and to mitigate the environmental and social consequences of waste disposal [114,115]. The application of emerging technologies and data analytics, such as BIM and big data, has been examined for CDW quantification, tracking, control, and management [107]. However, the utilization of these new digital technologies in CDW management is still in its infancy and decision-makers should be aware of the conditions that apply to information technology [29,116]. 

## 4. Research Gaps and Future Research Directions 

**Figure 7:**
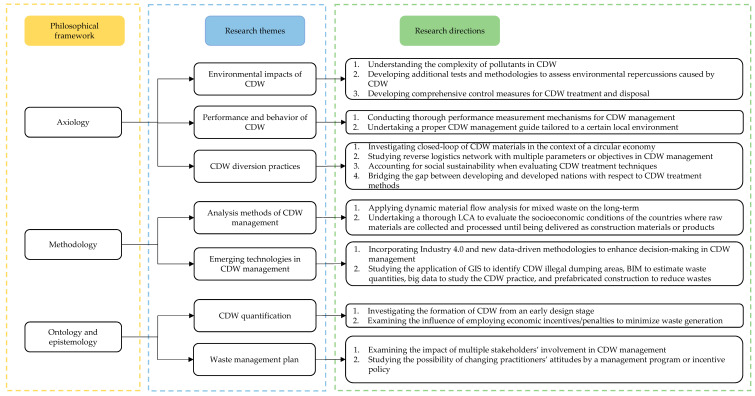
Research themes and directions in CDW management.

As illustrated in Figure 7, this section seeks to identify relevant future research directions based on a review of mainstream research themes and gaps as follows:

1. Understanding the environmental consequences of CDW is still inadequate, and few control measures for these pollutants have been developed [117]. As a result, future directions could include: (1) attempting to comprehend the complexity of pollutants in CDW, (2) developing additional tests and methodologies to assess environmental repercussions caused by CDW, and (3) developing comprehensive control measures for CDW treatment and disposal.
2. There have been some widely adopted management programs or incentive policies (e.g., landfilling charging rate) that aim at promoting CDW diversion. However, the effectiveness of these programs or policies has not been widely investigated [118]. Therefore, more thorough performance measurement mechanisms for CDW management, as well as a proper CDW management guide tailored to a certain local environment, are needed.
3. Closed loop of CDW materials should be further investigated in the context of a circular economy. This implies that waste materials shall be reused and recycled as resources in other life cycles, rather than being disposed of in landfills [119]. The reverse logistics network with uncertainties in multiple parameters (e.g., quality of recycled products, recycling rate and cost, and demand and supply rates) or objectives (i.e., social, environmental, and economic benefits) will also be a significant direction in regional CDW management.
4. There has been an insufficient focus on social sustainability when evaluating CDW treatment techniques, with a focus dedicated to the economic and environmental aspects of recycling CDW [120]. As a result, future research should focus on developing a technique that incorporates a framework, indicators, categories, and assessment indices for evaluating social sustainability. Furthermore, more studies are needed to perform comprehensive economic, social, and environmental assessments of CDW diversion practices.
5. Developing countries may encounter difficulties when they strive to learn from the adopted CDW treatment methods in developed countries. There has not been enough research to bridge the gap between developing and developed nations yet [121]. In this context, comparing the economic, social, and managerial aspects of CDW diversion practices would benefit the global research and practice community in determining appropriate CDW management plans in a particular country.
6. There is a research gap in the use of material flow analysis for mixed waste, which comprises many components with varying life periods and distributions, making data collection and analysis more complicated. Therefore, extending the combination of numerous data collecting sources, methodologies, and data processing techniques to research many materials is advisable. Additionally, it is recommended to conduct more research on assessing the environmental and economic implications of materials stocks and flows in the long term [122].
7. For LCA applications, the existing literature focuses on specific cities without tracing the primary source of materials. A thorough LCA should evaluate the socioeconomic conditions of the countries where raw materials are collected and processed until being delivered as construction materials or products. When selecting the best end-of-life option for CDW, the same considerations should be taken into account [123].
8. For the emerging technologies, future research can expand the application of geographic information systems (GIS) to identify CDW illegal dumping areas, BIM to estimate waste quantities, ensure waste management cooperation among stakeholders, and analyze waste throughout the building life cycle, big data to study the CDW practice, and prefabricated construction to reduce the generated waste [124,125,126,127]. There is also a need to incorporate Industry 4.0 and new data-driven methodologies (e.g., digital twin and artificial intelligence) to enhance decision-making in CDW management [123].
9. Most recent research has focused on waste disposal and treatment, with little effort made to avoid the formation of CDW from an early design stage [128]. The CDW generation is affected by multiple factors, such as stakeholder attitudes and behavior as well as economic incentives [129]. However, limited studies have been undertaken to examine the influence of employing economic incentives/penalties (e.g., disposal charging system) to minimize waste generation.
10. Human aspects shall be investigated in relation to the CDW management program [110]. There has been little research to date that examines the influence of multiple stakeholders’ involvement in CDW management. Therefore, further studies are required to understand ways to foster multi-sectoral engagement and collaborative governance [130,131]. The focus shall be also given to the possibility of changing practitioners’ attitudes through a management program or incentive policy as indicated by Yuan and Shen [132].

## 5. Discussion

This review-based study applied three methodological steps (i.e., bibliometric search, scientometric analysis, and qualitative discussion) to review CDW management. The bibliometric search incorporated searching for relevant publications in the Scopus database. These studies were further analyzed using scientometric analysis to identify the most significant journals, authors, publications, and active countries. The last step of the scientometric analysis comprised conducting co-occurrence and clustering analysis of keywords using Vosviewer software. It is worth mentioning that this software did not represent the influence degree of each cluster of keyword groups, and it instead dealt with each individual keyword. It is very challenging to generate meaningful statements from the abundance of keywords with the means of Vosviewer. However, it can be interpreted that the research themes could be classified into seven clusters, where each cluster is characterized by a unique color that is assigned by the software. The clusters are identified as follows: (1) waste quantification approaches (green cluster), (2) analysis methods of waste management (blue/ purple clusters), (3) environmental impacts of waste (orange cluster), (4) performance and behavior of waste (red cluster), (5) waste management plan (cyan color), (6) waste diversion practices (yellow cluster), and (7) emerging technologies in waste management (brown cluster).

This research undertook an in-depth qualitative assessment based on the four-fold philosophical framework to interpret the identified research topics. This affirmed the link between step 2 (cluster keyword in scientometric analysis) and step 3 (qualitative discussion) in the workflow of this research study. The four philosophical aspects of axiology, methodology, ontology, and epistemology were implicitly addressed in all of the keywords. The concept behind assigning the identified seven themes to the four aspects of the framework was described here. The functional dimension considered three themes: (1) associated environmental impacts, which studied the environmental implications of CDW management; (2) related performance and behavior, which investigated the performance and behavior of waste and recycled products; and (3) adopted diversion practices, which examined CDW disposal practices. The two themes that elaborated on the methodological component were: (1) analysis methods, which specified the research methodologies used for data collecting and analysis in the study of CDW management; and (2) developing technologies, which investigated the use of information technology in this field. Waste quantification, which involved estimating the volumes of accumulated and generated waste in order to adopt the appropriate waste management strategy, and waste management plan, which discussed waste minimization and prevention methods to ensure effective decision-making in the early project stages, were the two themes that described the ontological and epistemological dimensions. Categorization of the research themes based on the four dimensions aided in better understanding the research themes, highlighting current gaps, and providing a framework for future research directions.

## 6. Conclusions

This research applied a holistic approach of bibliometric search, scientometric analysis, and qualitative discussion to present the key characteristics and disciplines in construction and demolition waste (CDW) management research. Based on the bibliometric search, the Scopus database generated 996 publications from 2001 to 2021. By scanning over the titles and abstracts of these articles, 895 publications were identified. The scientometric analysis was applied to determine the time distribution, journals, scholars, citations, active countries, co-citations of references, and keywords. Finally, this study shed the light on the most recent findings in the field of CDW management research.

According to the findings, there had been a shift in the development and promotion of the CDW management field. Since 2006, the publishing output had increased rapidly, and it was distinguished by a multidisciplinary and multi-regional approach. The highest contributions to the research community came from Vietnam, Ireland, and Denmark. In terms of the emerging keywords, review, urbanization, resource recovery, waste recycling, and environmental assessment were the top five keywords. The study used qualitative analysis based on the four-fold philosophical framework to highlight the essential topics, state the current gaps, and make recommendations for future research in this domain. Estimation and quantification, comprehensive analysis and assessment, environmental impacts, performance and behavior tests, management plan, diversion practices, and emerging technologies in CDW management were the key emerging research topics. After examining the current gaps in the identified research areas, the proposed research directions included understanding the composition of different materials for CDW, undertaking a proper CDW management guide tailored to a certain local environment, investigating the closed loop of CDW materials in the context of a circular economy, applying dynamic material flow analysis for mixed waste on the long-term. In addition, future research studies might focus on incorporating new data-driven methodologies to enhance decision-making in CDW management, examining the influence of employing economic incentives/penalties to minimize waste generation, and examining the impact of multiple stakeholders’ involvement in CDW management.

This review article could aid scholars in identifying the most prominent journals and researchers for prospective cooperation or publishing opportunities. It also assisted in grasping contemporary trends and hotspots in order to have a thorough comprehension of the studied topic. Besides, practitioners can be guided in implementing best practices and identifying further business prospects in the CDW management area. The government could be also guided in developing appropriate policies to promote the development of a waste-free building and construction industry.

## Figures and Tables

**Table 1:** Quantitative analyses of the most influential journals in CDW management research.

Journal	Number of Publications	Average Publication Year	Total Citations	Average Citations	Average Normal Citations
Journal of cleaner production	71	2018.24	3133	44.13	2.2
Materials	7	2019.86	120	17.14	1.84
Resources, conservation and recycling	95	2014.04	7354	77.41	1.84
Construction and building materials	14	2016.36	845	60.36	1.81
Building and environment	8	2009.88	723	90.38	1.48
Waste management	129	2015.22	7522	58.31	1.48
Automation in construction	4	2017.5	165	41.25	1.44
Renewable and sustainable energy reviews	7	2014.57	444	63.43	1.44
Science of the total environment	10	2019.3	213	21.3	1.4
Journal of environmental management	8	2019	143	17.88	1.34

**Table 2:** Quantitative analyses of some researchers in CDW management research.

Researcher	Number of Publications	Average Citations	Average Publication Year	Total Citations	Average Normal Citations
Kabirifar K.	6	21.17	2020.67	127	5.61
Shen L.	9	114.33	2013.33	1029	2.66
Yuan H.	29	76.07	2015.28	2206	2.2
Zillante G.	7	46.29	2018.71	324	2.18
Bao Z.	11	19.64	2020.18	216	2.02
Chi B.	6	20.67	2020	124	1.74
Yu A.T.W.	16	77.44	2014.12	1239	1.7
Wu Z.	11	52.27	2017.91	575	1.7
Lu W.	43	41.74	2017.77	1795	1.62
Xue F.	9	4.33	2020.67	39	1.11

**Table 3:** Most influential publications in CDW management research.

Author	Title	Journal	Publication Year	Total Citations	Normal Citations
Liu et al. [59]	An environmental assessment model of construction and demolition waste based on system dynamics: A case study in Guangzhou	Environmental Science and Pollution Research	2020	97	8.58
Huang et al. [13]	Construction and demolition waste management in China through the 3R principle	Resources Conservation and Recycling	2018	245	7.82
Gálvez-Martos et al. [60]	Construction and demolition waste best management practice in Europe	Resources Conservation and Recycling	2018	219	6.99
Ghaffar et al. [61]	Pathways to circular construction: An integrated management of construction and demolition waste for resource recovery	Journal of cleaner production	2020	75	6.63
Ruiz et al. [62]	The circular economy in the construction and demolition waste sector-a review and an integrative model approach	Journal of cleaner production	2020	71	6.28
Verian et al. [63]	Properties of recycled concrete aggregate and their influence in new concrete production	Resources Conservation and Recycling	2018	191	6.1
Marinković et al. [58]	Comparative environmental assessment of natural and recycled aggregate concrete	Waste Management	2010	358	5.76
Liu et al. [64]	Exploring factors influencing construction waste reduction: A structural equation modeling approach	Journal of cleaner production	2020	64	5.66
Osmani et al. [57]	Architects’ perspectives on construction waste reduction by design	Waste Management	2008	259	5.59
Jin et al. [29]	Science mapping approach to assisting the review of construction and demolition waste management research published between 2009 and 2018	Resources Conservation and Recycling	2019	100	5.58

**Table 4:** Active countries in CDW management research.

Country	Number of Publications	Average Publication Year	Number of Citations	Average Citations	Average Normal Citations
Vietnam	9	2019.89	139	15.44	3.02
Ireland	4	2013.25	367	91.75	2.26
Denmark	8	2016.25	516	64.5	1.86
Singapore	7	2015	478	68.29	1.65
Serbia	4	2016.25	383	95.75	1.64
United States	47	2014.34	1699	36.15	1.58
Hong Kong	116	2014.91	6879	59.3	1.57
Australia	111	2017	3790	34.14	1.49
United Kingdom	75	2015.2	3451	46.01	1.4
China	189	2017.61	6624	35.05	1.36

**Table 5:** Co-citations of references in CDW management research.

Authors	Title	Journal	Publication Year	Total Citations
Huang et al. [13]	Construction and demolition waste management in China through the 3R principle	Resources Conservation and Recycling	2018	21
Jin et al. [72]	An empirical study of perceptions towards construction and demolition waste recycling and reuse in China	Resources Conservation and Recycling	2017	21
Marzouk and Azab [73]	Environmental and economic impact assessment of construction and demolition waste disposal using system dynamics	Resources Conservation and Recycling	2014	21
Li et al. [74]	A model for estimating construction waste generation index for building project in China	Resources Conservation and Recycling	2013	19
Ajayi et al. [75]	Waste effectiveness of the construction industry: Understanding the impediments and requisites for improvements	Resources Conservation and Recycling	2015	18

**Table 6:** Summary of the top five keywords in CDW management research.

Keyword	Occurrences	Average Publication Year	Average Citations	Average Normal Citations
Review	6	2017.83	66.17	2.44
Urbanization	5	2018.20	52.00	2.27
Resource recovery	6	2013.17	51.17	2.12
Waste recycling	11	2017.45	51.45	1.84
Environmental assessment	6	2019.67	22.00	1.81

## Data Availability

The data used in this study are available in the Supplementary Materials.

## Supplementary Materials

The following are available online at https://www.mdpi.com/article/10.3390/ijerph19084496/s1, articles related to construction and demolition waste management as retrieved from Scopus database between 2001 and 2021.
